# Meta-Analysis of Neoadjuvant Immunotherapy for Patients with Resectable Non-Small Cell Lung Cancer

**DOI:** 10.3390/curroncol28060395

**Published:** 2021-11-14

**Authors:** Christopher Cao, Anthony Le, Matthew Bott, Chi-Fu Jeffrey Yang, Dominique Gossot, Franca Melfi, David H. Tian, Allen Guo

**Affiliations:** 1 Department of Cardiothoracic Surgery, Royal Prince Alfred Hospital, Sydney University, Sydney, NSW 2050, Australia; anthony.le1@health.nsw.gov.au (A.L.); z5213592@student.unsw.edu.au (A.G.); 2 Chris O’Brien Lifehouse Hospital, Sydney, NSW 2050, Australia; 3 Thoracic Surgery Service, Memorial Sloan Kettering Cancer Center, New York, NY 10065, USA; bottm@mskcc.org; 4 Division of Thoracic Surgery, Massachusetts General Hospital, Boston, MA 02114, USA; cjyang@mgh.harvard.edu; 5 Department of Thoracic Surgery, Institut du Thorax Curie-Montsouris, 75014 Paris, France; dominique.gossot@imm.fr; 6 Robotic Multispecialty Center for Surgery Robotic, Minimally Invasive Thoracic Surgery, University of Pisa, 56124 Pisa, Italy; franca.melfi@unipi.it; 7 Department of Anaesthesia and Perioperative Medicine, Westmead Hospital, Sydney, NSW 2145, Australia; david.tian@health.nsw.gov.au

**Keywords:** immunotherapy, meta-analysis, non-small cell lung cancer, neoadjuvant therapy

## Abstract

Purpose: Immunotherapy has created a paradigm shift in the treatment of metastatic non-small cell lung cancer (NSCLC), overcoming the therapeutic plateau previously achieved by systemic chemotherapy. There is growing interest in the utility of immunotherapy for patients with resectable NSCLC in the neoadjuvant setting. The present systematic review and meta-analysis aim to provide an overview of the existing evidence, with a focus on pathological and radiological response, perioperative clinical outcomes, and long-term survival. Methods: A systematic review was conducted using electronic databases from their dates of inception to August 2021. Pooled data on pathological response, radiological response, and perioperative outcomes were meta-analyzed where possible. Results: Eighteen publications from sixteen studies were identified, involving 548 enrolled patients who underwent neoadjuvant immunotherapy, of whom 507 underwent surgery. Pathologically, 52% achieved a major pathological response, 24% a complete pathological response, and 20% reported a complete pathological response of both the primary lesion as well as the sampled lymph nodes. Radiologically, 84% of patients had stable disease or partial response. Mortality within 30 days was 0.6%, and morbidities were reported according to grade and frequency. Conclusion: The present meta-analysis demonstrated that neoadjuvant immunotherapy was feasible and safe based on perioperative clinical data and completion rates of surgery within their intended timeframe. The pathological response after neoadjuvant immunotherapy was superior to historical data for patients who were treated with neoadjuvant chemotherapy alone, whilst surgical and treatment-related adverse events were comparable. The limitations of the study included the heterogenous treatment regimens, lack of long-term follow-up, variations in the reporting of potential prognostic factors, and potential publication bias.

## 1. Introduction

The emergence of immune checkpoint inhibitors (ICIs) transformed the landscape of treatment pathways for patients with metastatic non-small cell lung cancer (NSCLC) after encouraging results were reported from randomized controlled trials [1]. For patients with resectable NSCLC, the therapeutic plateau achieved by systemic chemotherapy as an adjuvant treatment reported a modest improvement of 5% over five years [2]. In the context of favorable outcomes identified in the metastatic NSCLC population, there is growing enthusiasm for neoadjuvant immunotherapy in patients with resectable NSCLC. The proposed benefits of immunotherapy prescribed in the neoadjuvant setting include the increased release of neoantigens from the tumor to stimulate the expansion of specific T-cells, enhanced control of micro-metastases, and enabling the assessment of biologic and immunologic responses of the tumor from resected specimens [3].

Due to the relative paucity of robust clinical data, there is an urgent need to assess the existing literature to analyze the feasibility, safety, and efficacy of neoadjuvant immunotherapy. The primary aims of the present systematic review and meta-analysis were to identify the pathological and radiological response rates of neoadjuvant ICIs. Secondary endpoints included perioperative mortality, surgical morbidity, treatment-related adverse events, delays in surgery, and the overall long-term and disease-free survival outcomes.

## 2. Materials and Methods

### 2.1. Search Strategy

Our methods adhered to the guidelines set forth in the Preferred Reporting Items for Systematic Review and Meta-Analyses: The PRISMA Statement. A systematic review was performed using online databases from their dates of inception to August 2021, including EMBASE, Ovid Medline, and all EBM Reviews. Search terms included neoadjuvant* and (“NSCLC” or “carcinoma, non-small cell lung” or “Non small cell lung”) and (“surg*” or “resect*” or “lobectomy” or “VATS” or “thoracic surgery, video-assisted”) as either Medical Subject Headings or keywords. Reference lists of all retrieved full texts were screened for further identification of potentially relevant studies.

### 2.2. Selection Criteria and Data Extraction

Selected studies included those in which patients with histologically proven NSCLC were treated with ICI prior to surgical resection and provided data on radiological and pathological response. Publications were limited to human subjects and written in English. Case studies involving 10 or fewer patients, conference abstracts, and poster presentations were excluded. Two investigators (A.G. and A.L.) independently reviewed each retrieved article. Discrepancies between the two reviewers were resolved by discussion and consensus after review by the senior investigator (C.C.).

### 2.3. Statistical Analysis

Meta-analysis of proportions or means was performed for categorical or continuous variables via generalized linear mixed models, as appropriate [4]. A random-effects model was applied to account for differing local surgical and immunotherapy protocols. Pooled data are presented as N (%) with 95% confidence intervals (CI). I^2^ statistic was used to estimate the percentage of total variation across studies due to heterogeneity rather than chance. Thresholds for I^2^ values for low, moderate, and high heterogeneity were considered as 0–49%, 50–74% and ≥75%, respectively. Specific analyses considering confounding factors were not possible because raw data were not available. All *p*-values were 2-sided, and ≤0.05 were considered statistically significant. All statistical analyses were conducted with Review Manager Version 5.1.2 (Cochrane Collaboration, Software Update, Oxford, UK) or R Version 4.0.2 (R Foundation for Statistical Computing, Vienna, Austria).

## 3. Results

### 3.1. Quantity and Quality of Trials

A total of 4143 references were identified through the electronic search; 2914 potentially relevant articles remained for screening after the removal of duplicated studies. After applying the selection criteria, 33 studies remained for full assessment, and 18 publications from 16 studies were selected for quantitative analysis [5,6,7,8,9,10,11,12,13,14,15,16,17,18,19,20,21,22]. Two publications reported on the same trials with a focus on different clinical outcomes [6,7,12,13]. Eleven publications from nine studies were prospectively registered in national clinical trial registries [5,6,7,8,9,10,11,12,13,14,15]. There was one randomized controlled trial, which compared neoadjuvant nivolumab with nivolumab and ipilimumab prior to surgical resection [8]. Neoadjuvant immunotherapy agents included durvalumab, nivolumab, ipilimumab, pembrolizumab, atezolizumab, sintilimab, and camrelizumab. A summary of the search strategy is presented in the PRISMA chart in Supplementary Figure S1, and a summary of the study characteristics is presented in Table 1.

### 3.2. Patient Characteristics

In total, 548 patients were treated with at least one cycle of neoadjuvant immunotherapy, with 507 patients (96%) undergoing subsequent surgery. The overall incidence of male patients was 73.7%, and the interquartile range of age across different studies was 61.5–65.5. Overall, 81.7% of patients were either former or current smokers. Histologically, 56.6% of patients had squamous cell carcinoma, 36.9% had adenocarcinoma, and 4.2% had other subtypes. The clinical stage was reported according to either the 7th or 8th edition of the TNM staging system, with 78.0% of patients reported as clinical stage IIIA and 1.0% of patients as stage IIIB [23,24]. Further details of patient characteristics are summarized in Table 2.

### 3.3. Surgical Approach and Resection Type

The most common type of resection was lobectomy (67.5%), followed by bilobectomy (12.1%), and pneumonectomy (8.6%). Surgical access was performed with minimal invasiveness through a video-assisted or robotic-assisted approach in 47.4% of operations, but 12.4% patients underwent a conversion to open thoracotomy after an intended minimally invasive approach. Overall, thoracotomy was performed in 51.7% of all operations. A complete microscopic resection (R0) was reported in 97.3% of all patients. The interquartile time interval from the final dose of immunotherapy to the time of operation was 27–32 days, and 2.0% of patients were delayed from their intended time of operation after treatment with neoadjuvant immunotherapy. The interquartile range of operative duration was 171–239 min. A total of 11 transfusion events occurred in 417 patients (6.9%). A summary of surgical details is presented in Table 3.

### 3.4. Radiological Response

**Figure 1:**
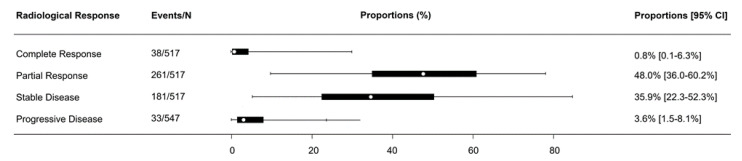
Forest plot summarizing the proportion of patients with radiological response after neoadjuvant immunotherapy for resectable non-small cell lung cancer.

Radiological response outcomes were consistently reported according to the Response Evaluation Criteria in Solid Tumors (RECIST) criteria [25]. Overall, 0.8% (95% confidence interval (CI): 0.1–6.3%) of patients reported complete response, 48.0% (95% CI: 36.0–60.2%) reported partial response, 35.9% (95% CI: 22.3–52.3%) reported stable disease, and 3.6% (95% CI: 1.5–8.1%) reported progressive disease, as presented in Figure 1.

### 3.5. Pathological Response

**Figure 2:**
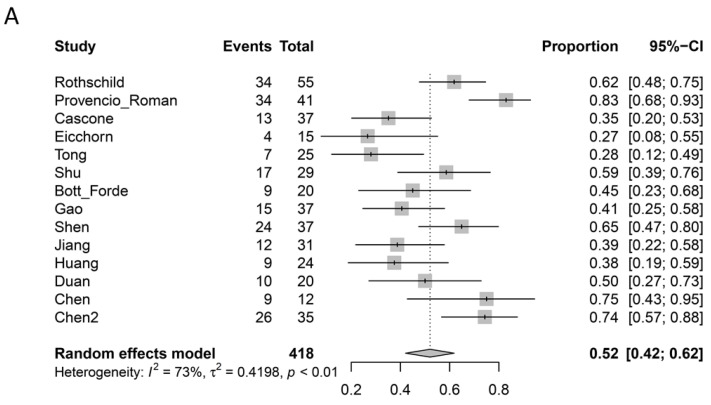

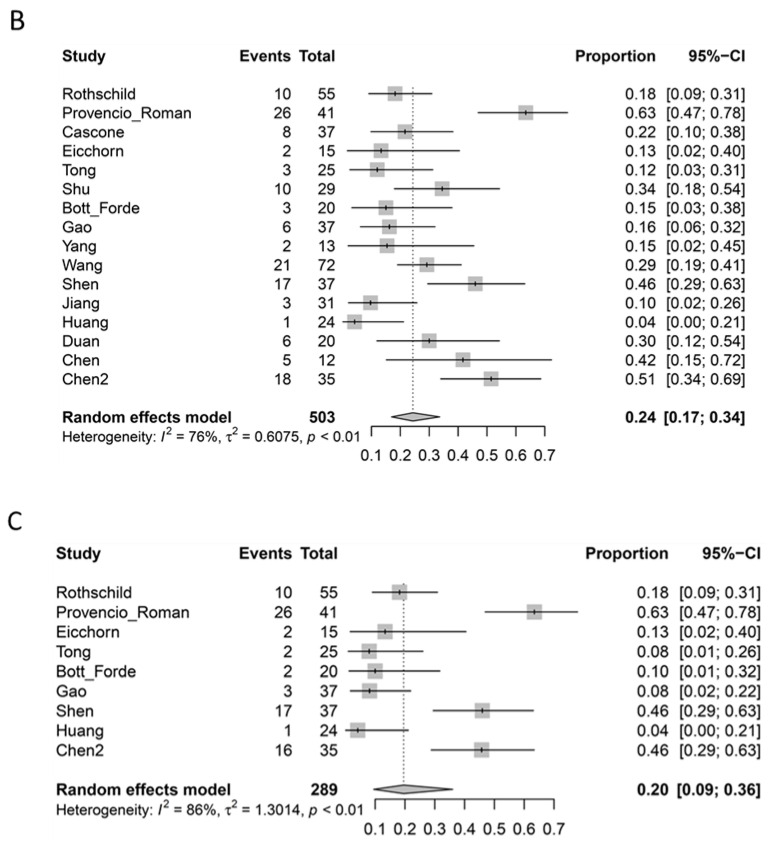
(**A**) Forest plot summarizing the proportion of patients with major pathological responses after neoadjuvant immunotherapy for resectable non-small cell lung cancer. (**B**) Forest plot summarizing the proportion of patients with complete pathological response of the primary tumor after neoadjuvant immunotherapy for resectable non-small cell lung cancer. (**C**) Forest plot summarizing the proportion of patients with complete pathological response of the primary tumor and sampled lymph nodes after neoadjuvant immunotherapy for resectable non-small cell lung cancer.

Pathological response outcomes were reported as ‘major pathological response’ (MPR) when less than 10% of the viable tumor was identified in the primary lesion, and ‘complete pathological response’ (pCR) when no viable tumor was identified. However, some studies specifically reported pCR when both the primary lesion as well as the sampled lymph nodes were free from any viable tumor [5,6,7,9,10,12,13,14,17,19,22], whereas others did not specify if nodal assessments were performed for pathological responses [8,11,15,16,18,20,21]. In addition, two studies defined MPR and pCR as being mutually exclusive, whereby patients who achieved a complete pathological response were not included within the group defined as a major pathological response [9,21]. The pathological response data from these studies were adjusted during statistical analysis to conform with other reports that included pCR patients within the MPR group. From the available data, 52% (95% CI: 42–62%; I^2^ = 73%) of patients who underwent surgery following neoadjuvant immunotherapy achieved MPR, 24% (95% CI, 17–34%; I^2^ = 76%) achieved pCR of the primary lesion, and 20% (95% CI: 9–36%; I^2^ = 86%) achieved pCR of both the primary lesion as well as the sampled lymph nodes. A summary of radiological and pathological response rates is presented in Table 4, and meta-analyzed forest plots of MPR, pCR, and pCR, including lymph nodes, are presented in Figure 2A–C.

### 3.6. Mortality and Morbidity

Overall, four deaths (0.6%) were reported within 30 days of surgery from all selected studies. However, some studies reported deaths within the same admission beyond 30 days [5]. Adverse events were commonly reported according to the grades of severity, ranging from grade 1–5. The most common surgical complications included prolonged air leak, pneumonia, atrial arrhythmias, chylothorax, and recurrent laryngeal nerve injury. The most common treatment-related adverse events included fatigue, anorexia, nausea, alopecia, neutropenia, and rash. A summary of surgical and treatment-related adverse events, including specified grade 3–5 adverse events, are summarized in Supplementary Tables S1 and S2, respectively, and illustrated in Supplementary Figures S2 and S3. Adverse events were only tabulated if they were reported in three or more individual studies, unless the severity of an adverse event was ≥3, in which case they were included irrespective of frequency.

### 3.7. Overall Survival and Disease-Free Survival

Six studies provided survival data in the form of Kaplan–Meier graphs, but a statistical summary of these data was not possible due to different timeframes of survival calculation [5,6,8,11,12,22]. Survival was calculated from the time of registration [5], diagnosis [6], randomization [8], treatment initiation [11], surgery [13], or unspecified reasons [22]. The follow-up periods of these studies were also limited, ranging from 13–29 months.

## 4. Discussion

The present systematic review and meta-analysis aimed to provide an overview of the existing evidence for patients who underwent neoadjuvant immunotherapy for resectable NSCLC. The key findings of the study identified a major pathological response rate of 52% and a complete pathological response of 24%. These values compared favorably to historical data for chemotherapy, which reported estimated rates of MPR and pCR as 22% and 4%, respectively [26,27]. When the sampled lymph nodes as well as the primary lesions were assessed by selected studies, the meta-analysis of pCR for neoadjuvant immunotherapy was 20%. The radiological response was less consistent, with 83.9% of patients reporting either stable disease or partial regression according to RECIST criteria. The lack of correlation between pathological and radiological responses can be partially attributed to the pseudoprogression phenomenon, whereby the infiltration of T-cells and peritumoral inflammation were associated with the increased size and activity of lesions on imaging, but favorable pathological responses in histopathology [12]. The incidence of this phenomenon in the recent NEOSTAR and NEOMUN trials was low, and its clinical significance remains to be seen [6,7,9]. The present study demonstrated the feasibility and safety of immune checkpoint inhibitors when given prior to surgery, with 96% of patients undergoing surgery after systemic treatment, and a surgical delay rate of 2.0%. The overall 30-day mortality rate was 0.6% across all studies, and surgical morbidities were similar in type and frequency to contemporary series of thoracic resections without neoadjuvant immunotherapy [28,29].

Surgical resection was performed by open thoracotomy in 51.7% of all cases, including in 12.4% patients who were converted from an intended minimally invasive approach. These findings compared favorably to outcomes reported by the National Cancer Data Base, which reported a thoracotomy rate of 73.2% and a conversion rate of 18.9% for VATS and 10.3% for robotic VATS [30]. For patients with advanced-stage NSCLC who underwent neoadjuvant chemotherapy, outcomes from tertiary institutions reported conversion rates of 26% after attempted VATS or robotic VATS [29,31]. The higher proportion of patients who were able to complete their operations via a minimally invasive approach identified in the present systematic review was likely due to the selection of specialized academic centers recruited for clinical trials. Encouragingly, the completeness of resection (R0) was achieved in 97.3% of all patients, and the pneumonectomy rate was 8.6%, which was relatively low compared to other series that reported 15.8–17.6% for patents who had neoadjuvant chemotherapy [29,32]. Technical challenges after neoadjuvant immunotherapy included increased fibrosis, adhesions, and granulomatous changes found within lymph nodes that Cascone termed ‘nodal immune flare’ [8], which could make the dissection around critical structures difficult and unsafe. Similar findings were reported by Bott, who also described dense adhesions surrounding the fissure and aorta [12].

Several limitations should be acknowledged from the present study, and results should be interpreted with caution. Some endpoints were inconsistently reported by studies identified in the present systematic review. Most importantly, a complete pathological response was defined as ‘no viable tumor within the resected specimen’, but there was variable reporting on whether the resected lymph nodes were also assessed. Travis advocated for a systematic approach to evaluate sampled nodes, particularly in the context of clinical trials, to confirm an absence of a tumor within the nodes (ypN0) after neoadjuvant systemic therapy [33]. Several studies [10,12,13,14,22] reported the presence of a tumor in nodal specimens when the primary lesions had pCR, and future studies should routinely assess and report on the pathological response of nodal tissue to understand their incidence and clinical significance. The variations in patient inclusion criteria, neoadjuvant treatment regimen, and subsequent adjuvant therapy may impact the overall and disease-free survival outcomes, which should also be reported from well-defined timeframes, such as the time of operation. The follow-up periods were relatively short, and only limited survival data have been published to date. Finally, there is a potential publication bias, as the abstracts identified in our screening process reported the early termination of trials due to lack of efficacy or excessive postoperative mortality, but their data were not included for quantitative analysis as they did not meet the study selection criteria [34,35].

Many challenging questions remain about the utility of immunotherapy for patients with resectable NSCLC. The potential prognostic value of PD-L1 (programmed death-ligand 1) was evaluated at separate cut-off points and measured against different surrogate endpoints such as MPR and pCR [5,6,7,8,9,22]. Such variations between studies dilute the strength of data interpretation, and the impact on overall survival and justification for patient selection remains uncertain. Future studies evaluating PD-L1 should routinely report on standardized endpoints such as overall survival and pCR, with accepted thresholds such as <1% vs. >1%. The type of immune check inhibitor, number of cycles, and additional prescription of chemotherapy in both neoadjuvant and adjuvant settings varied between studies. The optimal treatment regimen is most likely personalized to the individual patient based on predictive factors not yet elucidated from the published data. Larger studies with longer follow-up may answer some of these questions, and novel predictors of response, such as microbiome analysis and tumor mutational burden should be further examined [8,13].

## Figures and Tables

**Table 1:** Study characteristics of trials on neoadjuvant immunotherapy for patients with resectable non-small cell lung cancer.

Study	Institution	Recruitment Period	F/U (Months)	Immunotherapy	Chemotherapy	Adjuvant Immunotherapy
Rothschild, 2021 [5]	14 institutions in Sweden	6/2016–1/2019	29	Durvalumab (750 mg) 2 cycles	Cisplatin + docetaxel	Durvalumab 26 cycles
NADIM Provencio, 2021 [6] Roman, 2021 [7]	18 institutions in Spain	4/2017–8/2018	24	Nivolumab (360 mg) 3 cycles	Paclitaxel + carboplatin 3 cycles	Nivolumab (240 mg q2w for 4 months then 480 mg q4w for 8 months)
NEOSTAR Cascone, 2021 [8]	MD Anderson Cancer Center, USA	6/2017–11/2018	22	Nivolumab (3 mg/kg on D1, 15, 29) 3 cycles or Nivolumab 3 cycles + Ipilimumab (1 mg/kg on D1 only)	NS	NS
NEOMUN Eichhorn, 2021 [9]	Heidelberg University Hospital, Germany	5/2018–3/2020	NS	Pembrolizumab (200 mg) 2 cycles	NS	NS
Tong, 2021 [10]	Mayo Clinic; Dartmouth- Hitchcock; Duke University, USA	4/2017–2/2019	11	Pembrolizumab (200 mg) 2 cycles	NS	Pembrolizumab 4 cycles
Shu, 2020 [11]	Columbia University; MGH; BWH; Vanderbilt University Medical Center, USA	5/2016–3/019	13	Atezolizumab (1200 mg) 4 cycles	Paclitaxel + carboplatin 4 cycles	NS
Bott, 2019 [12] Forde, 2018 [13]	Johns Hopkins; MSKCC, USA	8/2015–10/2016	20	Nivolumab (3 mg/kg) 2 cycles	NS	NS
Gao, 2020 [14]	PUMC	3/2018–3/2019	3	Sintilimab (200 mg) 2 cycles	NS	Sintilimab
Yang, 2018 [15]	Duke University Medical Centre, USA	3/2013–12/2015	24	Ipilimumab (10 mg/kg) 2 cycles	Paclitaxel + cisplatin or carboplatin 3 cycles	NS
Wang, 2021 [16]	Zhejiang Cancer Hospital, China	9/2019–7/2020	NS	Nivolumab (200 mg), pembrolizumab (100 mg), camrelizumab (200 mg) 2 cycles	Paclitaxel + carboplatin q3w	NS
Shen, 2021 [17]	Zhejiang Cancer Hospital, China	6/2019–7/2020	7	Pembrolizumab (2 mg/kg) 2 cycles	Paclitaxel + carboplatin 2 cycles	NS
Jiang, 2021 [18]	Shanghai Chest Hospital, China	9/2018–4/2020	NS	Pembrolizumab or nivolumab 3 cycles	NS	Variable
Huang, 2021 [19]	Qingdao University Hospital, China	6/2019–12/2020	NS	Nivolumab (3 mg/kg) 2 cycles	NS	NS
Duan, 2021 [20]	Tangdu Hospital; Chongqing Medical University, China	6/2018–6/2020	NS	Sintilimab or nivolumab or pembrolizumab, 3–4 cycles	Pemetrexed + cisplatin or Paclitaxel + nedaplatin or Gemcitabine + nedaplatin or Paclitaxel + Carboplatin 3–4 cycles	NS
Chen, 2021 [21]	Shanghai Chest Hospital, China	1/2019–3/2020	18	Pembrolizumab 4 cycles or nivolumab 2 cycles	Carboplatin and paclitaxel	Variable
Chen, 2021 [22]	Tianjin Medical University Cancer Institute and Hospital	1/2019–5/2020	13	Pembrolizumab (2 mg/kg) 2 cycles q3w	Cisplatin + paclitaxel liposome or pemetrexed q3w	NS

MSKCC, Memorial Sloan Kettering Cancer Centre; PUMC, Peking Union Medical College; MGH, Massachusetts General Hospital; BWH, Brigham and Women’s Hospital; F/U, Follow-up; NS, Not specified.

**Table 2:** A summary of baseline patient characteristics in selected studies on neoadjuvant immunotherapy for resectable non-small cell lung cancer.

									Histopathology			Clinical Stage					
Study	Neoadjuvant Immunotherapy	Operation (%)		Male (%)		Age	Smoking History (%)		SCC	ADC	Other	IA	IB	IIA	IIB	IIIA	IIIB
Rothschild [5]	62 ^	55	88.7%	35	52.2%	61	64	92.3%	22	37	8	0	0	0	0	67	0
Provencio [6] * Roman [7] *	46	41	89.1%	34	73.9%	63	46	100%	16	26	4	0	0	0	1	45	0
Cascone [8] *	44	39	88.6%	28	63.6%	65.6	36	81.8%	17	26	1	8	15	7	5	9	0
Eicchorn [9] *	15	15	100%	7	46.7%	59.8	-	-	2	13	0	0	0	0	6	9	0
Tong [10]	30	25	83.3%	16	53.3%	72	26	86.7%	17	10	3	0	9	7	6	8	0
Shu [11]	30	29	96.7%	15	50.0%	67	30	100%	12	17	1	0	0	4	3	23	0
Bott [12] Forde [13]	22	20	90.9%	10	45.5%	67	18	81.8%	5	14	2	2	2	5	5	7	0
Gao [14] *	40	39	97.5%	33	82.5%	62	32	80.0%	33	6	1	2	6	1	13	10	8
Yang [15]	24	13	54.2%	12	50.0%	65	23	95.8%	9	15	0	0	0	3	2	19	0
Wang [16]	72	72	100%	66	91.7%	62.2	60	83.3%	66	5	1	0	0	0	0	72	0
Shen [17]	37	37	100%	35	94.6%	62.8	31	83.8%	37	0	0	0	0	0	3	28	6
Jiang [18] *	31	31	100%	29	93.5%	61	7	22.6%	22	9	0	0	0	1	4	16	10
Huang [19] *	25	24	96.0%	16	64.0%	62.9	15	60.0%	8	13	3	0	0	0	0	25	0
Duan [20]	23	20	87.0%	22	95.7%	61.8	22	95.7%	19	4	0	0	0	3	3	8	9
Chen [21] *	12	12	100%	9	75.0%	61	9	75.0%	4	6	2	0	0	0	0	7	5
Chen [22] *	35	35	100%	29	82.9%	62.2	27	77.1%	26	7	2	0	0	0	0	31	4
Total	548	507	95.9%	396	73.7%	IQR (61.5–65.5)	446	81.7%	56.6%	36.9%	4.2%	0.1%	0.2%	2.3%	6.2%	78.0%	1.0%

* AJCC 8th edition TNM staging system; ^ 62/67 enrolled patients received neoadjuvant immunotherapy; SCC, squamous cell carcinoma; ADC, adenocarcinoma.

**Table 3:** A summary of operative details for patients who underwent neoadjuvant immunotherapy in the treatment of resectable non-small cell lung cancer.

	Resection Margin			Type of Surgery							Surgical Approach			Final Immunotherapy to Surgery			Blood Loss	
Study	R0	R1	R2	Pneumonectomy	Bilobectomy	Lobectomy	Sleeve Lobectomy	Wedge	Other	Exploratory	Thoracotomy	MIS	Conversion to Open	Median Days	Delay (n)	Time (min)	Blood Loss (mL)	Transfusion
Rothschild [5]	51	3	1	5	7	43	-	-	-	-	-	-	-	-	-	-	-	-
Provencio [6], Roman [7]	41	0	0	3	3	32	3	0	-	0	24/41	17/41	4/41	-	0	195	-	1
Cascone [8]	39	0	0	-	-	-	-	-	-	-	-	-	-	31	8	-	-	-
Eicchorn [9]	15	0	0	0	0	15	0	0	-	0	-	-	-	-	1	-	-	-
Tong [10]	22	3	0	3	1	18	2	-	1	-	7/25	18/25	5/25	26	1	305	-	2
Shu [11]	26	-	-	3	4	19	0	0	-	3	14/29	12/29	-	27	0	-	-	2
Bott [12] Forde [13]	20	-	-	2	1	15	1	1	-	-	14/20	6/20	7/20	18	0	228	100	-
Gao [14]	36	0	1	13	5	18	1	0	-	2	29/39	10/39	-	-	2	-	-	-
Yang [15]	13	0	0	1	1	10	0	1	-	-	4/13	9/13	3/13	-	2	-	-	2
Wang [16]	-	-	-	-	-	-	-	-	-	-	-	-	-	-	0	-	-	-
Shen [17]	37	0	0	2	7	22	6	-	-	-	12/37	25/37	-	-	-	184	-	-
Jiang [18]	24	4	3	2	4	18	7	0	0	0	23/31	8/31	1	34	-	158	200	2
Huang [19]	23	1	0	1	3	19	-	-	1	-	0/24	24/24	-	29	0	196	92	-
Duan [20]	19	1	0	2	2	11	5	0	-	0	6/20	14/20	2/20	-	-	250	212.5	2
Chen [21]	12	0	0	0	1	8	3	0	-	0	9/12	3/12	-	28	1	140	200	-
Chen [22]	35	0	0	3	9	9	-	-	14 ^#^	-	34/35	1/35	-	33	0	-	-	-
Overall	97.3%	1.7%	0.6%	8.6%	12.1%	67.5%	7.8%	0.9%	5.0%	1.4%	51.7%	47.4%	12.4%	IQR (27– 32)	2.0%	IQR (171–239)	96–207	6.9%

Duration from last dose of immunotherapy; ^#^ included sleeve and Pancoast tumor resections; IQR, Interquartile range; MIS, minimally invasive surgery.

**Table 4:** A summary of radiological and pathological responses after neoadjuvant immunotherapy for patients with resectable non-small cell lung cancer.

	Radiological Response *				Pathological Response		
Study	CR	PR	SD	PD	Major Pathological Response	Complete Pathological Response Primary Lesion	Complete Pathological Response Primary Lesion + Nodes
Rothschild [5]	4/62	32/62	16/62	7/62	34/55	10/55	10/55
Provencio [6] Roman [7]	2/46	33/46	11/46	0	34/41	26/41	26/41
Cascone [8]	1/44	8/44	28/44	6/44	13/37	8/37	-
Eicchorn [9]	0	4/15	10/15	0	4/15 ^	2/15	2/15
Tong [10]	-	-	-	1/30	7/25	3/25	2/25
Shu [11]	0	19/30	9/30	2/30	17/29	10/29	-
Bott [12] Forde [13]	0	2/21	18/21	1/21	9/20	3/20	2/20
Gao [14]	0	8/40	28/40	4/40	15/37	6/37	3/37
Yang [15]	0	14/24	2/24	8/24	-	2/13	-
Wang [16]	21/72	47/72	3/72	1/72	-	21/72	-
Shen [17]	10/37	22/37	5/37	0	24/37	17/37	17/37
Jiang [18]	0	24/31	5/31	2/31	12/31	3/31	-
Huang [19]	0	8/25	16/25	1/25	9/24	1/24	1/24
Duan [20]	0	17/23	6/23	0	10/20	6/20	-
Chen [21]	0	6/12	6/12	0	9/12 ^	5/12	-
Chen [22]	0	17/35	18/35	0	26/35	18/35	16/35
Total	**0.8%**	**48.0%**	**35.9%**	**3.6%**	**52.0%**	**24.3%**	**19.6%**

* According to Response Evaluation Criteria in Solid Tumors (RECIST) criteria: CR, complete response; PR, partial response; SD, stable disease; PD, progressive disease. ^ Major pathological response included all patients with <10% viable tumor.

## Supplementary Materials

The following are available online at https://www.mdpi.com/article/10.3390/curroncol28060395/s1, Figure S1: PRIMSA flow chart detailing the literature search process for studies on neoadjuvant immunotherapy and surgery for non-small cell lung cancer; Figure S2: Post-surgical adverse events for patients with resectable non-small cell lung cancer after neoadjuvant immunotherapy; Figure S3: Treatment-related adverse events for patients with resectable non-small cell lung cancer after neoadjuvant systemic therapy; Table S1: Postoperative surgical adverse events of patients who underwent neoadjuvant immunotherapy and resection for non-small cell lung cancer; Table S2 A summary of treatment-related adverse events for patients who underwent neoadjuvant immunotherapy for resectable non-small cell lung cancer.

## Abbreviations

ICI	immune checkpoint inhibitor
NSCLC	non-small cell lung cancer
CI	confidence intervals
VATS	video assisted thoracoscopic surgery
RECIST	response evaluation criteria in solid tumors
MPR	major pathological response
pCR	complete pathological response
PD-L1	programmed death-ligand 1
