# Supplementary material for: Meta-Analysis of Neoadjuvant Immunotherapy for Patients with Resectable Non-Small Cell Lung Cancer
Source: Curr Oncol. 2021 Nov 14;28(6):4686–701. doi: 10.3390/curroncol28060395 (PMC8628782; doi:10.3390/curroncol28060395)
Supplement: Supplementary file 1 [file curroncol-28-00395-s001.zip › curroncol-1427800-supplementary.pdf]

Current Oncology

Meta-Analysis of Neoadjuvant Immunotherapy for Patients with Resectable Non-Small Cell Lung Cancer

Christopher Cao, Anthony Le, Matthew Bott, Jeffrey Yang, Dominique Gossot, Franca Melfi, David H. Tian and Allen Guo

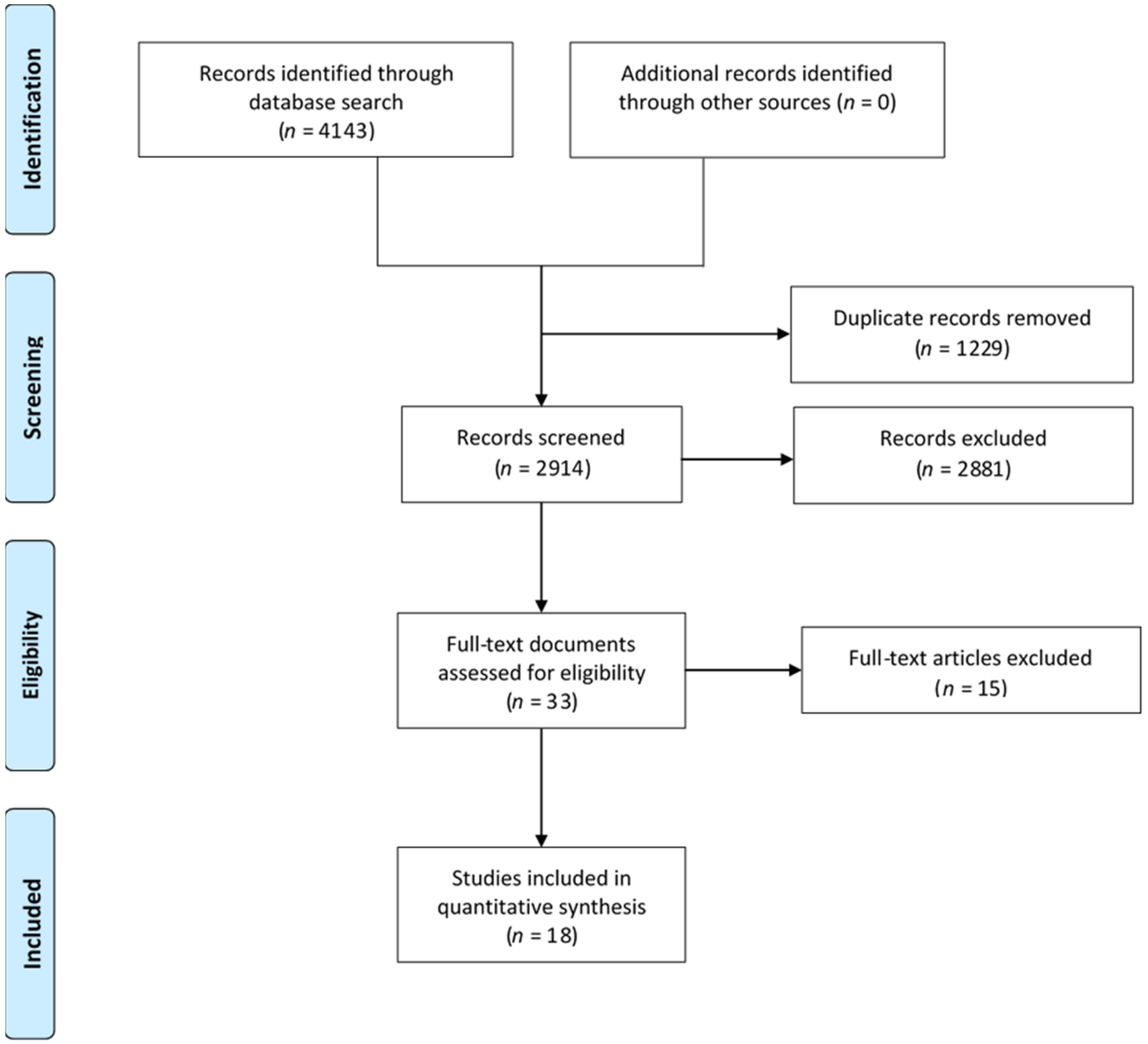

Figure S1. PRIMSA flow chart detailing the literature search process for studies on neoadjuvant immunotherapy and surgery for non-small cell lung cancer.

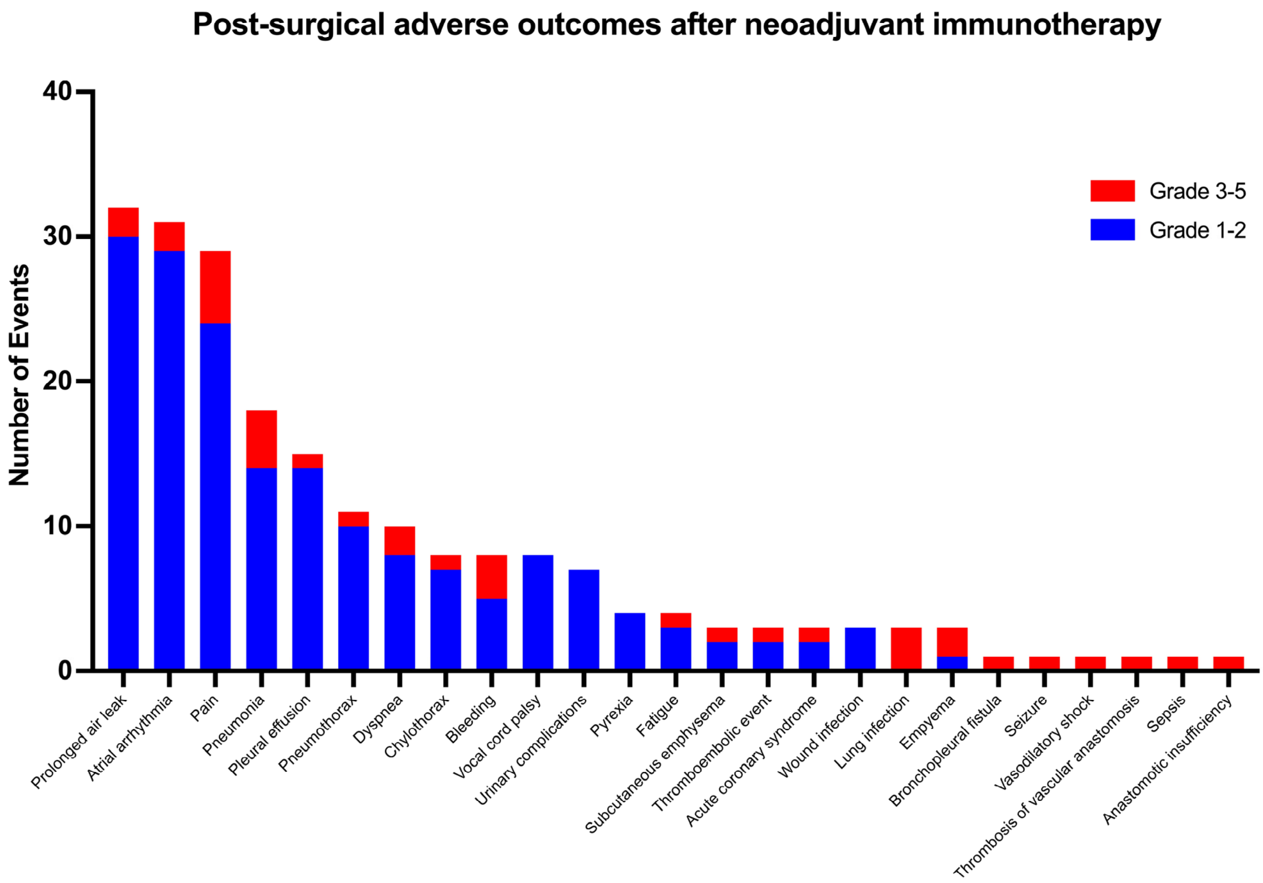

Figure S2. Post-surgical adverse events for patients with resectable non-small cell lung cancer after neoadjuvant immunotherapy.

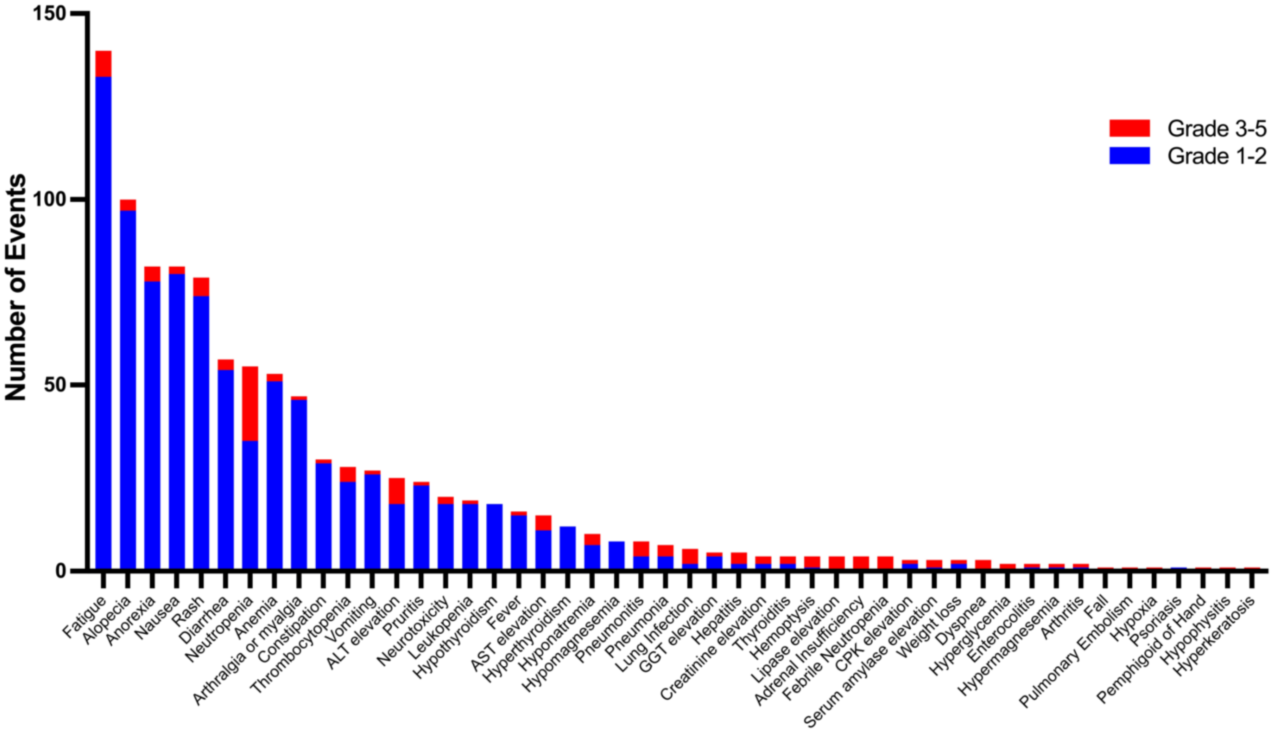

Figure S3. Treatment-related adverse events for patients with resectable non-small cell lung cancer after neoadjuvant systemic therapy.

Table S1. Postoperative surgical adverse events of patients who underwent neoadjuvant immunotherapy and resection for non-small cell lung cancer.

	Rothschild[5]	Provencio[6] Romano[7]	Cascone[8]	Eicchorn[9]	Tong[10]	Shu[11]	Bott[12] Forde[13]	Gao[14]	Yang[15]	Wang[16]	Shen[17]	Jiang[18]	Huang[19]	Duan[20]	Chen[21]	Chen[22]
**30-day mortality**	1	0	0	0	0	1	0	2	0	0	0	0	0	0	0	0
**HLOS (days)**	-	5	-	-	4	4	4	-	5	-	-	7	5.8	11	-	-
**Pneumonia**	-	5	1*	1	1	1*	1^	1*	0	4	2	1	-	-	-	-
**Prolonged air leak**	1^	8	-	-	4	-	1	-	2	-	-	14	1^	-	1	-
**Atrial arrhythmia**	-	4	-	-	6	3	6	-	1^	5	2	3	1^	0	-	-
**Chylothorax**	1^	1	-	-	-	-	-	-	-	3	1	1	-	-	1	-
**Vocal cord palsy**	1	1	-	-	-	-	-	-	1	2	2	-	-	1	-	-
**Pain**	26^	1	-	-	-	-	-	1	1	-	-	-	-	-	-	-
**Bleeding**	3^*	2	-	-	-	-	-	-	-	-	2	-	-	-	1	-
**Urinary complications**	-	-	-	-	-	2	1	-	3	-	-	-	-	1	-	-
**Pleural effusion**	4^	-	-	-	-	-	-	-	-	8	3	-	-	-	-	-
**Subcutaneous emphysema**	-	1	-	-	1	-	-	-	-	-	-	-	1^	-	-	-
**Thromboembolic event**	-	-	-	-	1	-	1	-	1^	-	-	-	-	-	-	-
**Acute coronary syndrome**	1^	-	-	-	-	-	1	-	1	-	-	-	-	-	-	-
**Pyrexia**	2	-	-	-	-	-	-	1	1	-	-	-	-	-	-	-
**Wound infection**	-	-	-	-	1	-	-	1	-	-	-	1	-	-	-	-
**Pneumothorax**	2^	-	-	-	-	-	-	-	-	-	9	-	-	-	-	-
**Empyema**	1^	-	1*	-	-	-	1	-	-	-	-	-	-	-	-	-
**Dyspnea**	10^	-	-	-	-	-	-	-	-	-	-	-	-	-	-	-
**Fatigue**	4^	-	-	-	-	-	-	-	-	-	-	-	-	-	-	-
**Lung infection**	3^	-	-	-	-	-	-	-	-	-	-	-	-	-	-	-
**Bronchopleural fistula**	-	-	1*	-	-	-	-	-	-	-	-	-	-	-	-	-
**Seizure**	-	-	-	-	-	-	-	1*	-	-	-	-	-	-	-	-
**Vasodilatory shock**	-	-	-	-	-	-	-	-	1^	-	-	-	-	-	-	-
**Anastomosis complication**	1^	1^	-	-	-	-	-	-	-	-	-	-	-	-	-	-
**Sepsis**	1*	-	-	-	-	-	-	-	-	-	-	-	-	-	-	-

^Grade 3-4 Adverse Event; *Grade 5 Adverse Event; HLOS, Hospital length of stay; Tabulated adverse events were limited to those reported in 3 or more studies, or if the adverse outcome was grade ≥3.

Table S2. A summary of treatment-related adverse events for patients who underwent neoadjuvant immunotherapy for resectable non-small cell lung cancer.

		Rothschild[5]	Provencio[6] Romano[7]	Cascone[8]	Eicchorn[9]	Tong[10]	Shu[11]	Bott[12] Forde[13]	Gao[14]	Yang[15]	Wang[16]	Shen[17]	Jiang[18]	Huang[19]	Duan[20]	Chen[21]	Chen[22]
	Anemia	-	7	5	-	-	21*	-	-	4	9*	4	-	-	3	-	-
	ALT elevation	4*	5*	3	-	<10%	6*	1	2	-	-	4*	-	-	-	-	-
**Hematologic**	AST elevation	3*	-	1	-	<10%	5*	-	2	-	-	4	-	-	-	-	-
	GGT elevation	-	-	-	-	-	-	-	5*	-	-	-	-	-	-	-	-
	CPK elevation	-	-	-	-	-	-	-	3*	-	-	-	-	-	-	-	-

	Lipase elevation	1*	3*	-	-	-	-	-	-	-	-	-	-	-	-	-	-
	Neutropenia	-	4*	-	-	-	26*	-	-	-	10*	8*	-	-	7	-	-
	Thrombocytopenia	-	-	-	-	-	19*	-	-	-	6*	3*	-	-	-	-	-
	Leukopenia	-	-	-	-	-	-	-	-	-	12*	-	-	-	7	-	-
	Creatinine elevation	-	3*	1	-	-	-	-	-	-	-	-	-	-	-	-	-
	Serum amylase elevation	-	3*	-	-	-	-	-	-	-	-	-	-	-	-	-	-
	Febrile neutropenia	-	3*	-	-	-	1*	-	-	-	-	-	-	-	-	-	-
**Gastrointestinal**	Nausea	9*	15	8	-	<10%	13	-	2	1	29*	5	-	-	-	-	-
	Vomiting	-	8	2	-	-	5	2	-	1	-	-	-	-	9*	-	-
	Constipation	-	8	-	-	-	7	-	-	-	9*	5	-	-	1	-	-
	Diarrhea	12*	11	9*	-	7	9*	2	-	-	-	4	-	-	2	1	-
	Enterocolitis	-	-	-	1	-	-	-	-	-	-	-	-	1*	-	-	-
	Hepatitis	1*	-	-	-	-	-	-	-	-	-	-	-	4*	-	-	-
**Systemic**	Fatigue	24*	24*	15	-	5	17*	-	-	1	39*	5	-	-	10*	-	-
	Weight loss	-	-	-	-	-	2*	-	-	1	-	-	-	-	-	-	-
	Anorexia	-	9*	1	-	<10%	3	3	-	1*	51*	6	-	-	8*	-	-
	Fever	-	-	1	-	-	3	1	6	-	5*	-	-	-	-	-	-
	Arthralgia or myalgia	8	21	2	-	3	5	-	-	-	-	6	-	-	2*	-	-
	Arthritis	2*	-	-	-	-	-	-	-	-	-	-	-	-	-	-	-
	Fall	-	-	1*	-	-	-	-	-	-	-	-	-	-	-	-	-
**Metabolic**	Hyperglycemia	-	-	-	-	<10%	1*	-	-	-	-	-	-	-	1*	-	-
	Hypermagnesemia	-	1	1*	-	-	-	-	-	-	-	-	-	-	-	-	-
	Hypomagnesemia	-	-	2	-	-	3	-	-	3	-	-	-	-	-	-	-
	Hyponatremia	-	-	5*	-	-	2*	-	2*	1	-	-	-	-	-	-	-
**Endocrine**	Adrenal insufficiency	1*	-	-	-	-	-	-	-	3*	-	-	-	-	-	-	-
	Hyperthyroidism	6	-	2	-	<10%	-	-	3	-	-	-	-	-	1	-	-
	Thyroiditis	-	-	-	4*	<10%	-	-	-	-	-	-	-	-	-	-	-
	Hypothyroidism	-	-	2	-	3	3	-	7	-	2	1	-	-	-	-	-
**Respiratory**	Hemoptysis	-	-	2*	-	-	-	-	2	-	-	-	-	-	-	-	-
	Pneumonitis	2*	-	2*	-	-	-	-	4*	-	-	-	-	-	-	-	-
	Lung infection	4*	-	-	-	-	-	-	2*	-	-	-	-	-	-	-	-
	Pulmonary embolism	-	-	1*	-	-	-	-	-	-	-	-	-	-	-	-	-
	Hypoxia	-	-	1*	-	-	-	-	-	-	-	-	-	-	-	-	-
	Dyspnea	3*	-	-	-	-	-	-	-	-	-	-	-	-	-	-	-
	Pneumonia	-	-	1*	-	-	-	1*	-	-	4*	-	-	1	-	-	-
**Skin**	Alopecia	-	17*	-	-	-	14	-	-	-	50*	9	-	-	10*	-	-
	Rash	9	11*	18	1*	4	5	-	2	-	20*	4	-	2	1	1	1*
	Pruritis	10*	7	4	-	3	-	-	-	-	-	-	-	-	-	-	-
	Psoriasis	-	-	1	-	<10%*	-	-	-	-	-	-	-	-	-	-	-
	Hyperkeratosis	1*	-	-	-	-	-	-	-	-	-	-	-	-	-	-	-
	Pemphigoid of hand	-	1*	-	-	-	-	-	-	-	-	-	-	-	-	-	-
**Neurological**	Hypophysitis	-	-	-	1*	-	-	-	-	-	-	-	-	-	-	-	-
	Neurotoxicity	-	15*	-	-	-	-	-	-	-	1	4	-	-	-	-	-

*At least 1 or more ≥ Grade 3 treatment related adverse event.
